# On Computability of Data Word Functions Defined by Transducers

**DOI:** 10.1007/978-3-030-45231-5_12

**Published:** 2020-04-17

**Authors:** Léo Exibard, Emmanuel Filiot, Pierre-Alain Reynier

**Affiliations:** 8 ENS/CNRS, Université Paris-Saclay, Cachan, France; 9 University of Duisburg-Essen, Duisburg, Germany; 10 Université Libre de Bruxelles, Brussels, Belgium; 11 Aix Marseille Univ, Université de Toulon, CNRS, LIS, Marseille, France

**Keywords:** Data Words, Register Automata, Register Transducers, Functionality, Continuity, Computability

## Abstract

In this paper, we investigate the problem of synthesizing computable functions of infinite words over an infinite alphabet (data $\omega$-words). The notion of computability is defined through Turing machines with infinite inputs which can produce the corresponding infinite outputs in the limit. We use non-deterministic transducers equipped with registers, an extension of register automata with outputs, to specify functions. Such transducers may not define functions but more generally relations of data $\omega$-words, and we show that it is PSpace-complete to test whether a given transducer defines a function. Then, given a function defined by some register transducer, we show that it is decidable (and again, PSpace-c) whether such function is computable. As for the known finite alphabet case, we show that computability and continuity coincide for functions defined by register transducers, and show how to decide continuity. We also define a subclass for which those problems are PTime.

## 1 Introduction

*Context* Program synthesis aims at deriving, in an automatic way, a program that fulfils a given specification. Such setting is very appealing when for instance the specification describes, in some abstract formalism (an automaton or ideally a logic), important properties that the program must satisfy. The synthesised program is then *correct-by-construction* with regards to those properties. It is particularly important and desirable for the design of safety-critical systems with hard dependability constraints, which are notoriously hard to design correctly.

Program synthesis is hard to realise for general-purpose programming languages but important progress has been made recently in the automatic synthesis

* A version with full proofs can be found at https://arxiv.org/abs/2002.08203.

** Funded by a FRIA fellowship from the F.R.S.-FNRS.

*** Research associate of F.R.S.-FNRS. Supported by the ARC Project Transform Fédération Wallonie-Bruxelles and the FNRS CDR J013116F; MIS F451019F projects.

† Partly funded by the ANR projects DeLTA (ANR-16-CE40-0007) and Ticktac (ANR-18-CE40-0015).

of *reactive systems*. In this context, the system continuously receives input signals to which it must react by producing output signals. Such systems are not assumed to terminate and their executions are usually modelled as infinite words over the alphabets of input and output signals. A specification is thus a set of pairs (in,out), where in and out are infinite words, such that out is a legitimate output for in. Most methods for reactive system synthesis only work for *synchronous* systems over *finite* sets of input and output signals $\Sigma$ and $\Gamma$. In this synchronous setting, input and output signals alternate, and thus *implementations* of such a specification are defined by means of *synchronous* transducers, which are Büchi automata with transitions of the form $(q, \sigma, \gamma, q')$, expressing that in state $q$, when getting input $\sigma \in \Sigma$, output $\gamma \in \Gamma$ is produced and the machine moves to state $q'$. We aim at building *deterministic* implementations, in the sense that the output $\gamma$ and state $q'$ uniquely depend on $q$ and $\sigma$. The realisability problem of specifications given as synchronous non-deterministic transducers, by implementations defined by synchronous deterministic transducers is known to be decidable [14,20]. In this paper, we are interested in the *asynchronous* setting, in which transducers can produce none or several outputs at once every time some input is read, i.e., transitions are of the form $(q, \sigma, w, q')$ where $w \in \Gamma^*$. However, such generalisation makes the realisability problem undecidable [2,9].

*Synthesis of Transducers with Registers* In the setting we just described, the set of signals is considered to be finite. This assumption is not realistic in general, as signals may come with unbounded information (e.g. process ids) that we call here *data*. To address this limitation, recent works have considered the synthesis of reactive systems processing *data words* [17,6,16,7]. Data words are infinite words over an alphabet $\Sigma \times \mathcal{D}$, where $\Sigma$ is a finite set and $\mathcal{D}$ is a possibly infinite countable set. To handle data words, just as automata have been extended to *register automata*, transducers have been extended to *register transducers*. Such transducers are equipped with a finite set of registers in which they can store data and with which they can compare data for equality or inequality. While the realisability problem of specifications given as synchronous non-deterministic register transducers ($\mathsf{NRT}_{\mathsf{syn}}$) by implementation defined by synchronous deterministic register transducers ($\mathsf{DRT}_{\mathsf{syn}}$) is undecidable, decidability is recovered for specifications defined by universal register transducers and by giving as input the number of registers the implementation must have [7,17].

*Computable Implementations* In the previously mentioned works, both for finite or infinite alphabets, implementations are considered to be deterministic transducers. Such an implementation is guaranteed to use only a constant amount of memory (assuming data have size $O(1)$). While it makes sense with regards to memory-efficiency, some problems turn out to be undecidable, as already mentioned: realisability of $\mathsf{NRT}_{\mathsf{syn}}$ specifications by $\mathsf{DRT}_{\mathsf{syn}}$, or, in the finite alphabet setting, when both the specification and implementation are asynchronous. In this paper, we propose to study computable implementations, in the sense of (partial) functions $f$ of data $\omega$-words computable by some Turing machine $M$ that has an infinite input $x \in \mathrm{dom}(f)$, and produces longer and longer prefixes of the output

$f(x)$ as it reads longer and longer prefixes of the input $x$. Therefore, such a machine produces the output $f(x)$ in the limit. We denote by $\mathsf{TM}$ the class of Turing machines computing functions in this sense. As an example, consider the function $f$ that takes as input any data $\omega$-word $u = (\sigma_1, d_1)(\sigma_2, d_2)\dots$ and outputs $(\sigma_1, d_1)^\omega$ if $d_1$ occurs at least twice in $u$, and otherwise outputs $u$. This function is not computable, as an hypothetic machine could not output anything as long as $d_1$ is not met a second time. However, the following function $g$ is computable. It is defined only on words $(\sigma_1, d_1)(\sigma_2, d_2)\dots$ such that $\sigma_1\sigma_2 \cdots \in ((a+b)c^*)^\omega$, and transforms any $(\sigma_i, d_i)$ by $(\sigma_i, d_1)$ if the next symbol in $\{a, b\}$ is an $a$, otherwise it keeps $(\sigma_i, d_i)$ unchanged. To compute it, a $\mathsf{TM}$ would need to store $d_1$, and then wait until the next symbol in $\{a, b\}$ is met before outputting something. Since the finite input labels are necessarily in $((a + b)c^*)^\omega$, this machine will produce the whole output in the limit. Note that $g$ cannot be defined by any deterministic register transducer, as it needs unbounded memory to be implemented.

However, already in the finite alphabet setting, the problem of deciding if a specification given as some non-deterministic synchronous transducer is realisable by some computable function is open. The particular case of realisability by computable functions of universal domain (the set of all $\omega$-words) is known to be decidable [12]. In the asynchronous setting, the undecidability proof of [2] can be easily adapted to show the undecidability of realisability of specifications given by non-deterministic (asynchronous) transducers by computable functions.

*Functional Specifications* As said before, a specification is in general a relation from inputs to outputs. If this relation is a function, we call it functional. Due to the negative results just mentioned about the synthesis of computable functions from non-functional specifications, we instead here focus on the case of functional specifications and address the following general question: given the specification of a function of data $\omega$-words, is this function "implementable", where we define "implementable" as "being computable by some Turing machine". Moreover, if it is implementable, then we want a procedure to automatically generate an algorithm that computes it. This raises another important question: how to decide whether a specification is functional ? We investigate these questions for asynchronous register transducers, here called register transducers. This asynchrony allows for much more expressive power, but is a source of technical challenge.

*Contributions* In this paper, we solve the questions mentioned before for the class of (asynchronous) non-deterministic register transducers ($\mathsf{NRT}$). We also give fundamental results on this class. In particular, we prove that:

1. deciding whether an $\mathsf{NRT}$ defines a function is PSpace-complete,
2. deciding whether two functions defined by $\mathsf{NRT}$ are equal on the intersection of their domains is PSpace-complete,
3. the class of functions defined by $\mathsf{NRT}$ is effectively closed under composition,
4. computability and continuity are equivalent notions for functions defined by $\mathsf{NRT}$, where continuity is defined using the classical Cantor distance,
5. deciding whether a function given as an $\mathsf{NRT}$ is computable is PSpace-c,

6. those problems are in PTime for a subclass of NRT, called test-free NRT.

Finally, we also mention that considering the class of deterministic register transducers (DRT for short) instead of computable functions as a yardstick for the notion of being "implementable" for a function would yield undecidability. Indeed, given a function defined by some NRT, it is in general undecidable to check whether this function is realisable by some DRT, by a simple reduction from the universality problem of non-deterministic register automata [19].

*Related Work* The notion of continuity with regards to Cantor distance is not new, and for rational functions over finite alphabets, it was already known to be decidable [21]. Its connection with computability for functions of $\omega$-words over a finite alphabet has recently been investigated in [3] for one-way and two-way transducers. Our results lift some of theirs to the setting of data words. The model of test-free NRT can be seen as a one-way non-deterministic version of a model of two-way transducers considered in [5].

## 2 Data Words and Register Transducers

For a (possibly infinite) set $S$, we denote by $S^*$ (resp. $S^\omega$) the set of finite (resp. infinite) words over this alphabet, and we let $S^\infty = S^* \cup S^\omega$. For a word $u = u_1 \dots u_n$, we denote $\|u\| = n$ its length, and, by convention, for $u \in S^\omega$, $\|u\| = \infty$. The empty word is denoted $\varepsilon$. For $1 \leq i \leq j \leq \|u\|$, we let $u[i{:}j] = u_i u_{i+1} \dots u_j$ and $u[i] = u[i{:}i]$ the $i$th letter of $u$. For $u, v \in S^\infty$, we say that $u$ is a prefix of $v$, written $u \preceq v$, if there exists $w \in S^\infty$ such that $v = uw$. In this case, we define $u^{-1}v = w$. For $u, v \in S^\infty$, we say that $u$ and $v$ *mismatch*, written $\mathsf{mismatch}(u, v)$, when there exists a position $i$ such that $1 \leq i \leq \|u\|$, $1 \leq i \leq \|v\|$ and $u[i] \neq v[i]$. Finally, for $u, v \in S^\infty$, we denote by $u \wedge v$ their longest common prefix, i.e. the longest word $w \in S^\infty$ such that $w \preceq u$ and $w \preceq v$.

*Data Words* In this paper, $\Sigma$ and $\Gamma$ are two finite alphabets and $\mathcal{D}$ is a countably infinite set of *data*. We use letter $\sigma$ (resp. $\gamma$, $d$) to denote elements of $\Sigma$ (resp. $\Gamma$, $\mathcal{D}$). We also distinguish an arbitrary data value $\mathsf{d}_0 \in \mathcal{D}$. Given a set $R$, let $\tau_0^R$ be the constant function defined by $\tau_0^R(r) = \mathsf{d}_0$ for all $r \in R$. Given a finite alphabet $A$, a *labelled data* is a pair $x = (a, d) \in A \times \mathcal{D}$, where $a$ is the *label* and $d$ the *data*. We define the projections $\mathsf{lab}(x) = a$ and $\mathsf{dt}(x) = d$. A *data word* over $A$ and $\mathcal{D}$ is an infinite sequence of labelled data, i.e. a word $w \in (A \times \mathcal{D})^\omega$. We extend the projections $\mathsf{lab}$ and $\mathsf{dt}$ to data words naturally, i.e. $\mathsf{lab}(w) \in A^\omega$ and $\mathsf{dt}(w) \in \mathcal{D}^\omega$. A *data word language* is a subset $L \subseteq (A \times \mathcal{D})^\omega$. Note that here, data words are infinite, otherwise they are called *finite data words.*

### 2.1 Register Transducers

Register transducers are transducers recognising data word relations. They are an extension of finite transducers to data word relations, in the same way register automata [15] are an extension of finite automata to data word languages. Here, we define them over infinite data words with a Büchi acceptance condition, and allow multiple registers to contain the same data, with a syntax close to [18]. The current data can be compared for equality with the register contents via tests, which are symbolic and defined via Boolean formulas of the following form. Given $R$ a set of registers, a *test* is a formula $\phi$ satisfying the following syntax:

$$\phi ::= \top \mid \bot \mid r^{=} \mid r^{\neq} \mid \phi \wedge \phi \mid \phi \vee \phi \mid \neg\phi$$

where $r \in R$. Given a valuation $\tau : R \to \mathcal{D}$, a test $\phi$ and a data $d$, we denote by $\tau, d \models \phi$ the satisfiability of $\phi$ by $d$ in valuation $\tau$, defined as $\tau, d \models r^{=}$ if $\tau(r) = d$ and $\tau, d \models r^{\neq}$ if $\tau(r) \neq d$. The Boolean combinators behave as usual. We denote by $\mathsf{Tst}_R$ the set of (symbolic) tests over $R$.

**Definition 1.** *A non-deterministic register transducer (* NRT *) is a tuple* $T = (Q, R, i_0, F, \Delta)$*, where* $Q$ *is a finite set of* states*,* $i_0 \in Q$ *is the* initial *state,* $F \subseteq Q$ *is the set of* accepting *states,* $R$ *is a finite set of* registers *and* $\Delta \subseteq Q \times \Sigma \times \mathsf{Tst}_R \times 2^R \times (\Gamma \times R)^* \times Q$ *is a finite set of* transitions. *We write* $q \xrightarrow[T]{\sigma,\phi|\mathsf{asgn},o} q'$ *for* $(q, \sigma, \phi, \mathsf{asgn}, o, q') \in \Delta$ *(*$T$ *is sometimes omitted).*

The semantics of a register transducer is given by a labelled transition system: we define $L_T = (C, \Lambda, \to)$, where $C = Q \times (R \to \mathcal{D})$ is the set of configurations, $\Lambda = (\Sigma \times \mathcal{D}) \times (\Gamma \times \mathcal{D})^*$ is the set of labels, and we have, for all $(q, \tau), (q', \tau') \in C$ and for all $(l, w) \in \Lambda$, that $(q, \tau) \xrightarrow{(l,w)} (q', \tau')$ whenever there exists a transition $q \xrightarrow[T]{\sigma,\phi|\mathsf{asgn},o} q'$ such that, by writing $l = (\sigma', d)$ and $w = (\gamma'_1, d_1) \dots (\gamma'_n, d_n)$:

- (Matching labels) $\sigma = \sigma'$
- (Compatibility) $d$ satisfies the test $\phi \in \mathsf{Tst}_R$, i.e. $\tau, d \models \phi$.
- (Update) $\tau'$ is the successor register configuration of $\tau$ with regards to $d$ and $\mathsf{asgn}$: $\tau'(r) = d$ if $r \in \mathsf{asgn}$, and $\tau'(r) = \tau(r)$ otherwise
- (Output) By writing $o = (\gamma_1, r_1) \dots (\gamma_m, r_m)$, we have that $m = n$ and for all $1 \leq i \leq n$, $\gamma_i = \gamma'_i$ and $d_i = \tau'(r_i)$.

Then, a *run* of $T$ is an infinite sequence of configurations and transitions $\rho = (q_0, \tau_0) \xrightarrow[L_T]{(u_1,v_1)} (q_1, \tau_1) \xrightarrow[L_T]{(u_2,v_2)} \cdots$. Its input is $\mathsf{in}(\rho) = u_1 u_2 \dots$, its output is $\mathsf{out}(\rho) = v_1 \cdot v_2 \dots$. We also define its sequence of states $\mathsf{st}(\rho) = q_0 q_1 \dots$, and its *trace* $\mathsf{tr}(\rho) = u_1 \cdot v_1 \cdot u_2 \cdot v_2 \dots$. Such run is *initial* if $(q_0, \tau_0) = (i_0, \tau_0^R)$. It is *final* if it satisfies the Büchi condition, i.e. $\inf(\mathsf{st}) \cap F \neq \varnothing$, where $\inf(\mathsf{st}) = \{q \in Q \mid q = q_i$ for infinitely many $i\}$. Finally, it is *accepting* if it is both initial and final. We then write $(q_0, \tau_0) \xrightarrow[T]{u|v}$ to express that there is a final run $\rho$ of $T$ starting from $(q_0, \tau_0)$ such that $\mathsf{in}(\rho) = u$ and $\mathsf{out}(\rho) = v$. In the whole paper, and unless stated otherwise, we always assume that the output of an accepting run is infinite ($v \in (\Gamma \times \mathcal{D})^\omega$), which can be ensured by a Büchi condition.

A *partial run* is a finite prefix of a run. The notions of input, output and states are extended by taking the corresponding prefixes. We then write $(q_0, \tau_0) \xrightarrow[T]{u|v}$

$(q_n, \tau_n)$ to express that there is a partial run $\rho$ of $T$ starting from configuration $(q_0, \tau_0)$ and ending in configuration $(q_n, \tau_n)$ such that $\mathsf{in}(\rho) = u$ and $\mathsf{out}(\rho) = v$.

Finally, the relation represented by a transducer $T$ is:

$$[\![T]\!] = \big\{(u, v) \in (\Sigma \times \mathcal{D})^\omega \times (\Gamma \times \mathcal{D})^\omega \mid \text{there exists an accepting run } \rho \text{ of } T \\ \text{such that } \mathsf{in}(\rho) = u \text{ and } \mathsf{out}(\rho) = v\big\}$$

*Example 2.* As an example, consider the register transducer $T_{\mathsf{rename}}$ depicted in Figure 1. It realises the following transformation: consider a setting in which we deal with logs of communications between a set of clients. Such a log is an infinite sequence of pairs consisting of a tag, chosen in some finite alphabet $\Sigma$, and the identifier of the client delivering this tag, chosen in some infinite set of data values. The transformation should modify the log as follows: for a given client that needs to be modified, each of its messages should now be associated with some new identifier. The transformation has to verify that this new identifier is indeed free, *i.e.* never used in the log. Before treating the log, the transformation receives as input the id of the client that needs to be modified (associated with the tag del), and then a sequence of identifiers (associated with the tag ch), ending with #. The transducer is non-deterministic as it has to guess which of these identifiers it can choose to replace the one of the client. In particular, observe that it may associate multiple output words to a same input if two such free identifiers exist.

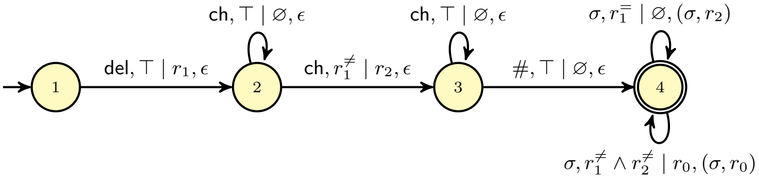

**Fig. 1.** A register transducer $T_{\mathsf{rename}}$. It has three registers $r_1$, $r_2$ and $r_0$ and four states. $\sigma$ denotes any letter in $\Sigma$, $r_1$ stores the id of del and $r_2$ the chosen id of ch, while $r_0$ is used to output the last data value read as input. As we only assign data to single registers, we write $r_i$ for the singleton assignment set $\{r_i\}$.

*Finite Transducers* Since we reduce the decision of continuity and functionality of NRT to the one of finite transducers, let us introduce them: a finite transducer (NFT for short) is an NRT with 0 registers (i.e. $R = \varnothing$). Thus, its transition relation can be represented as $\Delta \subseteq Q \times \Sigma \times \Gamma^* \times Q$. A direct extension of the construction of [15, Proposition 1] allows to show that:

**Proposition 3.** *Let $T$ be an* NRT *with $k$ registers, and let $X \subset_f \mathcal{D}$ be a finite subset of data. Then, $[\![T]\!] \cap (\Sigma \times X)^\omega \times (\Gamma \times X)^\omega$ is recognised by an* NFT *of exponential size, more precisely with $O(|Q| \times |X|^{|R|})$ states.*

## 2.2 Technical Properties of Register Automata

Although automata are simpler machines than transducers, we only use them as tools in our proofs, which is why we define them from transducers, and not the other way around. A non-deterministic register automaton, denoted NRA, is a transducer without outputs: its transition relation is $\Delta \subseteq Q \times \Sigma \times \mathsf{Tst}_R \times 2^R \times \{\varepsilon\} \times Q$ (simply represented as $\Delta \subseteq Q \times \Sigma \times \mathsf{Tst}_R \times 2^R \times Q$). The semantics are the same, except that now we lift the condition that the output $v$ is infinite since there is no output. For $A$ an NRA, we denote $L(A) = \{u \in (\Sigma \times \mathcal{D})^\omega \mid$ there exists an accepting run $\rho$ of $A$ over $u\}$. Necessarily the output of an accepting run is $\varepsilon$. In this section, we establish technical properties about NRA.

Proposition 4, the so-called "indistinguishability property", was shown in the seminal paper by Kaminski and Francez [15, Proposition 1]. Their model differs in that they do not allow distinct registers to contain the same data, and in the corresponding test syntax, but their result easily carries to our setting. It states that if an NRA accepts a data word, then such data word can be relabelled with data from any set containing $\mathsf{d}_0$ and with at least $k+1$ elements. Indeed, at any point of time, the automaton can only store at most $k$ data in its registers, so its notion of "freshness" is a local one, and forgotten data can thus be reused as fresh ones. Moreover, as the automaton only tests data for equality, their actual value does not matter, except for $\mathsf{d}_0$ which is initially contained in the registers.

Such "small-witness" property is fundamental to NRA, and will be paramount in establishing decidability of functionality (Section 3) and computability (Section 4). We use it jointly with Lemma 5, which states that the interleaving of the traces of runs of an NRT can be recognised with an NRA, and Lemma 6, which expresses that an NRA can check whether interleaved words coincide on some bounded prefix, and/or mismatch before some given position.

**Proposition 4 ([15]).** *Let $A$ be an* NRA *with $k$ registers. If $L(A) \neq \varnothing$, then, for any $X \subseteq \mathcal{D}$ of size $|X| \geq k+1$ such that $\mathsf{d}_0 \in X$, $L(A) \cap (\Sigma \times X)^\omega \neq \varnothing$.*

The runs of a register transducer $T$ can be flattened to their traces, so as to be recognised by an NRA. Those traces can then be interleaved, in order to be compared. The proofs of the following properties are straightforward.

Let $\rho_1 = (q_0, \tau_0) \xrightarrow[L_T]{(u_1, u_1')} (q_1, \tau_1) \dots$ and $\rho_2 = (p_0, \mu_0) \xrightarrow[L_T]{(v_1, v_1')} (p_1, \mu_1) \dots$ be two runs of a transducer $T$. Then, we define their *interleaving* $\rho_1 \otimes \rho_2 = u_1 \cdot u_1' \cdot v_1 \cdot v_1' \cdot u_2 \cdot u_2' \cdot v_2 \cdot v_2' \dots$ and $L_\otimes(T) = \{\rho_1 \otimes \rho_2 \mid \rho_1$ and $\rho_2$ are accepting runs of $T\}$.

**Lemma 5.** *If $T$ has $k$ registers, then $L_\otimes(T)$ is recognised by an* NRA *with $2k$ registers.*

**Lemma 6.** *Let $i, j \in \mathbb{N} \cup \{\infty\}$. We define $M_j^i = \{u_1 u_1' v_1 v_1' \cdots \mid \forall k \geq 1, u_k, v_k \in (\Sigma \times \mathcal{D}), u_k', v_k' \in (\Gamma \times \mathcal{D})^*, \forall 1 \leq k \leq j, v_k = u_k$ and $\|u_1' \cdot u_2' \cdots \wedge v_1' \cdot v_2' \dots\| \leq i\}$. Then, $M_j^i$ is recognisable by an* NRA *with $2$ registers and with $1$ register if $i = \infty$.*

## 3 Functionality, Equivalence and Composition of NRT

In general, since they are non-deterministic, NRT may not define functions but relations, as illustrated by Example 2. In this section, we first show that deciding whether a given NRT defines a function is PSPACE-complete, in which case we call it *functional*. We show, as a consequence, that testing whether two functional NRT define two functions which coincide on their common domain is PSPACE-complete. Finally, we show that functions defined by NRT are closed under composition. This is an appealing property in transducer theory, as it allows to define complex functions by composing simple ones.

*Example 7.* As explained before, the transducer $T_{\mathsf{rename}}$ described in Example 2 is not functional. To gain functionality, one can reinforce the specification by considering that one gets at the beginning a list of $k$ possible identifiers, and that one has to select the first one which is free, for some fixed $k$. This transformation is realised by the register transducer $T_{\mathsf{rename2}}$ depicted in Figure 2 (for $k = 2$).

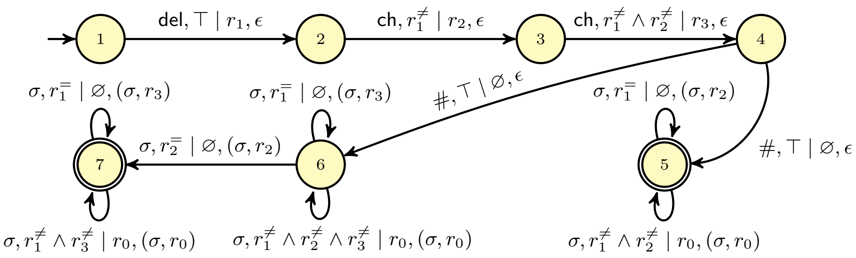

**Fig. 2.** A NRT $T_{\mathsf{rename2}}$, with four registers $r_1, r_2, r_3$ and $r_0$ (the latter being used, as in Figure 1, to output the last read data). After reading the # symbol, it guesses whether the value of register $r_2$ appears in the suffix of the input word. If not, it goes to state 5, and replaces occurrences of $r_1$ by $r_2$. Otherwise, it moves to state 6, waiting for an occurrence of $r_2$, and replaces occurrences of $r_1$ by $r_3$.

Let us start with the functionality problem in the data-free case. It is already known that checking whether an NFT over $\omega$-words is functional is decidable [13,11]. By relying on the pattern logic of [10] designed for transducers of *finite* words, it can be shown that it is decidable in NLOGSPACE.

**Proposition 8.** *Deciding whether an* NFT *is functional is in* NLOGSPACE.

The following theorem shows that a relation between data-words defined by an NRT with $k$ registers is a function iff its restriction to a set of data with at most $2k + 3$ data is a function. As a consequence, functionality is decidable as it reduces to the functionality problem of transducers over a finite alphabet.

**Theorem 9.** *Let $T$ be an* NRT *with $k$ registers. Then, for all $X \subseteq \mathcal{D}$ of size $|X| \geq 2k + 3$ such that $\mathsf{d}_0 \in X$, we have that $T$ is functional if and only if $[\![T]\!] \cap ((\Sigma \times X)^\omega \times (\Gamma \times X)^\omega)$ is functional.*

*Proof.* The left-to-right direction is trivial. Now, assume $T$ is not functional. Let $x \in (\Sigma \times \mathcal{D})^\omega$ be such that there exists $y, z \in (\Gamma \times \mathcal{D})^\omega$ such that $y \neq z$ and $(x, y), (x, z) \in [\![T]\!]$. Let $i = \|y \wedge z\|$. Then, consider the language $L = \{\rho_1 \otimes \rho_2 \mid \rho_1$ and $\rho_2$ are accepting runs of $T$, $\mathsf{in}(\rho_1) = \mathsf{in}(\rho_2)$ and $\|\mathsf{out}(\rho_1) \wedge \mathsf{out}(\rho_2)\| \leq i\}$. Since, by Lemma 5, $L_\otimes(T)$ is recognised by an $\mathsf{NRA}$ with $2k$ registers and, by Lemma 6, $M^i_\infty$ is recognised by an $\mathsf{NRA}$ with 2 registers, we get that $L = L_\otimes(T) \cap M^i_\infty$ is recognised by an $\mathsf{NRA}$ with $2k+2$ registers.

Now, $L \neq \varnothing$, since, by letting $\rho_1$ and $\rho_2$ be the runs of $T$ both with input $x$ and with respective outputs $y$ and $z$, we have that $w = \rho_1 \otimes \rho_2 \in L$. Let $X \subseteq \mathcal{D}$ such that $|X| \geq 2k+3$ and $\mathsf{d}_0 \in X$. By Proposition 4, we get that $L \cap (\Sigma \times X)^\omega \neq \varnothing$. By letting $w' = \rho'_1 \otimes \rho'_2 \in L \cap (\Sigma \times X)^\omega$, and $x' = \mathsf{in}(\rho'_1) = \mathsf{in}(\rho'_2)$, $y' = \mathsf{out}(\rho'_1)$ and $z' = \mathsf{out}(\rho'_2)$, we have that $(x', y'), (x', z') \in [\![T]\!] \cap ((\Sigma \times X)^\omega \times (\Gamma \times X)^\omega)$ and $\|y' \wedge z'\| \leq i$, so, in particular, $y' \neq z'$ (since both are infinite words). Thus, $[\![T]\!] \cap ((\Sigma \times X)^\omega \times (\Gamma \times X)^\omega)$ is not functional. □

As a consequence of Proposition 8 and Theorem 9, we obtain the following result. The lower bound is obtained by encoding non-emptiness of register automata, which is PSpace-complete [4].

**Corollary 10.** *Deciding whether an* $\mathsf{NRT}$ $T$ *is functional is* PSpace*-complete.*

Hence, the following problem on the equivalence of $\mathsf{NRT}$ is decidable:

**Theorem 11.** *The problem of deciding, given two functions $f, g$ defined by* $\mathsf{NRT}$*, whether for all $x \in \mathrm{dom}(f) \cap \mathrm{dom}(g)$, $f(x) = g(x)$, is* PSpace*-complete.*

*Proof.* The formula $\forall x \in \mathrm{dom}(f) \cap \mathrm{dom}(g) \cdot f(x) = g(x)$ is true iff the relation $f \cup g = \{(x, y) \mid y = f(x) \vee y = g(x)\}$ is a function. The latter can be decided by testing whether the disjoint union of the transducers defining $f$ and $g$ defines a function, which is in PSpace by Corollary 10. To show the hardness, we similarly reduce the emptiness problem of $\mathsf{NRA}$ $A$ over finite words, just as in the proof of Corollary 10. In particular, the functions $f_1$ and $f_2$ defined in this proof (which have the same domain) are equal iff $L(A) = \varnothing$. □

Note that under the promise that $f$ and $g$ have the same domain, the latter theorem implies that it is decidable to check whether the two functions are equal. However, checking $\mathrm{dom}(f) = \mathrm{dom}(g)$ is undecidable, as the language-equivalence problem for non-deterministic register automata is undecidable, since, in particular, universality is undecidable [19].

Closure under composition is a desirable property for transducers, which holds in the data-free setting [1]. We show that it also holds for functional $\mathsf{NRT}$.

**Theorem 12.** *Let $f, g$ be two functions defined by* $\mathsf{NRT}$*. Then, their composition $f \circ g$ is (effectively) definable by some* $\mathsf{NRT}$*.*

*Proof (Sketch).* By $f \circ g$ we mean $f \circ g : x \mapsto f(g(x))$. Assume $f$ and $g$ are defined by $T_f = (Q_f, R_f, q_0, F_f, \Delta_f)$ and $T_g = (Q_g, R_g, p_0, F_g, \Delta_g)$ respectively. Wlog we assume that the input and output finite alphabets of $T_f$ and $T_g$ are all equal to $\Sigma$, and that $R_f$ and $R_g$ are disjoint. We construct $T$ such that $[\![T]\!] = f \circ g$. The proof is similar to the data-free case where the composition is shown via a product construction which simulates both transducers in parallel, executing the second on the output of the first. Assume $T_g$ has some transition

$p \xrightarrow{\sigma,\phi|\{r\},o} q$ where $o \in (\Sigma \times R_g)^*$. Then $T$ has to be able to execute transitions of $T_f$ while processing $o$, even though $o$ does not contain any concrete data values (it is here the main important difference with the data-free setting). However, if $T$ knows the equality types between $R_f$ and $R_g$, then it is able to trigger the transitions of $T_f$. For example, assume that $o = (a, r_g)$ and assume that the content of $r_g$ is equal to the content of $r_f$, $r_f$ being a register of $T_f$, then if $T_f$ has some transition of the form $p' \xrightarrow{a,r_f^=|\{r'_f\},o'} q'$ then $T$ can trigger the transition $(p,q) \xrightarrow{\sigma,\phi|\{r\}\cup\{r'_f:=r_g\},o'} (p',q')$ where the operation $r'_f := r_g$ is a syntactic sugar on top of NRT that intuitively means "put the content of $r_g$ into $r'_f$". □

*Remark 13.* The proof of Theorem 12 does not use the hypothesis that $f$ and $g$ are functions, and actually shows a stronger result, namely that relations defined by NRT are closed under composition.

## 4 Computability and Continuity

We equip the set of (finite or infinite) data words with the usual distance: for $u, v \in (\Sigma \times \mathcal{D})^\omega$, $d(u,v) = 0$ if $u = v$ and $d(u,v) = 2^{-\|u \wedge v\|}$ otherwise. A sequence of (finite or infinite) data words $(x_n)_{n\in\mathbb{N}}$ converges to some infinite data word $x$ if for all $\epsilon > 0$, there exists $N \geq 0$ such that for all $n \geq N$, $d(x_n, x) \leq \epsilon$.

In order to reason with computability, we assume in the sequel that the infinite set of data values $\mathcal{D}$ we are dealing with has an effective representation. For instance, this is the case when $\mathcal{D} = \mathbb{N}$.

We now define how a Turing machine can compute a function of data words. We consider deterministic Turing machines, which three tapes: a read-only one-way input tape (containing the infinite input data word), a two-way working tape, and a write-only one-way output tape (on which it writes the infinite output data word). Consider some input data word $x \in (\Sigma \times \mathcal{D})^\omega$. For any integer $k \in \mathbb{N}$, we let $M(x,k)$ denote the output written by $M$ on its output tape after having read the $k$ first cells of the input tape. Observe that as the output tape is write-only, the sequence of data words $(M(x,k))_{k\geq 0}$ is non-decreasing.

**Definition 14 (Computability).** *A function $f : (\Sigma \times \mathcal{D})^\omega \to (\Gamma \times \mathcal{D})^\omega$ is computable if there exists a deterministic multi-tape machine $M$ such that for all $x \in \mathrm{dom}(f)$, the sequence $(M(x,k))_{k\geq 0}$ converges to $f(x)$.*

**Definition 15 (Continuity).** *A function $f : (\Sigma \times \mathcal{D})^\omega \to (\Gamma \times \mathcal{D})^\omega$ is* continuous *at $x \in \mathrm{dom}(f)$ if (equivalently):*

*(a) for all sequences of data words $(x_n)_{n\in\mathbb{N}}$ converging towards $x$, where for all $i \in \mathbb{N}$, $x_i \in \mathrm{dom}(f)$, we have that $(f(x_n))_{n\in\mathbb{N}}$ converges to $f(x)$.*
*(b) $\forall i \geq 0, \exists j \geq 0, \forall y \in \mathrm{dom}(f), \|x \wedge y\| \geq j \Rightarrow \|f(x) \wedge f(y)\| \geq i$.*

*Then, $f$ is continuous if and only if it is continuous at each $x \in \mathrm{dom}(f)$. Finally, a functional NRT $T$ is* continuous *when $[\![T]\!]$ is continuous.*

*Example 16.* We give an example of a non-continuous function $f$. The finite input and output alphabets are unary, and are therefore ignored in the description of $f$. Such function associates with every sequence $s = d_1 d_2 \cdots \in \mathcal{D}^\omega$ the word $f(s) = d_1^\omega$ if $d_1$ occurs infinitely many times in $s$, otherwise $f(s) = s$ itself.

The function $f$ is not continuous. Indeed, by taking $d \neq d'$, the sequence of data words $d(d')^n d^\omega$ converges to $d(d')^\omega$, while $f(d(d')^n d^\omega) = d^\omega$ converges to $d^\omega \neq f(d(d')^\omega) = d(d')^\omega$.

Moreover, $f$ is realisable by some $\mathsf{NRT}$ which non-deterministically guesses whether $d_1$ repeats infinitely many times or not. It needs only one register $r$ in which to store $d_1$. In the first case, it checks whether the current data $d$ is equal the content $r$ infinitely often, and in the second case, it checks that this test succeeds finitely many times, using Büchi conditions.

One can show that the register transducer $T_{\mathsf{rename2}}$ considered in Example 7 also realises a function which is not continuous, as the value stored in register $r_2$ may appear arbitrarily far in the input word. One could modify the specification to obtain a continuous function as follows. Instead of considering an infinite log, one considers now an infinite sequence of finite logs, separated by \$ symbols. The register transducer $T_{\mathsf{rename3}}$, depicted in Figure 3, defines such a function.

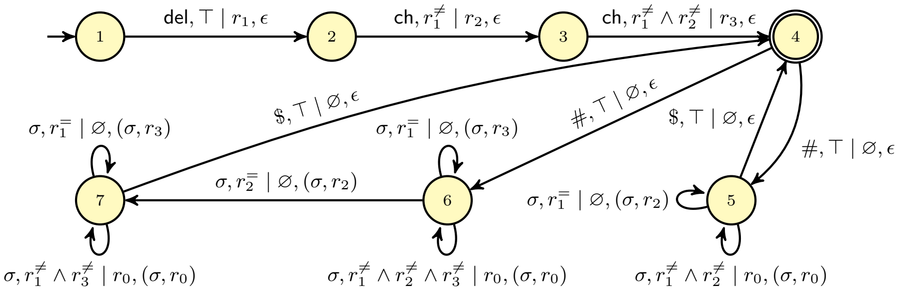

**Fig. 3.** A register transducer $T_{\mathsf{rename3}}$. This transducer is non-deterministic, yet it defines a continuous function.

We now prove the equivalence between continuity and computability for functions defined by $\mathsf{NRT}$. One direction, namely the fact that computability implies continuity, is easy, almost by definition. For the other direction, we rely on the following lemma which states that it is decidable whether a word $v$ can be safely output, only knowing a prefix $u$ of the input. In particular, given a function $f$, we let $\hat{f}$ be the function defined over all finite prefixes $u$ of words in $\mathrm{dom}(f)$ by $\hat{f}(u) = \bigwedge(f(uy) \mid uy \in \mathrm{dom}(f))$, the longest common prefix of all outputs of continuations of $u$ by $f$. Then, we have the following decidability result:

**Lemma 17.** *The following problem is decidable. Given an* $\mathsf{NRT}$ $T$ *defining a function* $f$*, two finite data words* $u \in (\Sigma \times \mathcal{D})^*$ *and* $v \in (\Gamma \times \mathcal{D})^*$*, decide whether* $v \preceq \hat{f}(u)$*.*

**Theorem 18.** *Let $f$ be a function defined by some* NRT $T$*. Then $f$ is continuous iff $f$ is computable.*

*Proof.* $\Leftarrow$ Assuming $f = [\![T]\!]$ is computable by some Turing machine $M$, we show that $f$ is continuous. Indeed, consider some $x \in \mathrm{dom}(f)$, and some $i \geq 0$. As the sequence of finite words $(M(x,k))_{k \in \mathbb{N}}$ converges to $f(x)$ and these words have non-decreasing lengths, there exists $j \geq 0$ such that $|M(x,j)| \geq i$. Hence, for any data word $y \in \mathrm{dom}(f)$ such that $|x \wedge y| \geq j$, the behaviour of $M$ on $y$ is the same during the first $j$ steps, as $M$ is deterministic, and thus $|f(x) \wedge f(y)| \geq i$, showing that $f$ is continuous at $x$.

$\Rightarrow$ Assume that $f$ is continuous. We describe a Turing machine computing $f$; the corresponding algorithm is formalised as Algorithm 1. When reading a finite prefix $x[{:}j]$ of its input $x \in \mathrm{dom}(f)$, it computes the set $P_j$ of all configurations $(q, \tau)$ reached by $T$ on $x[{:}j]$. This set is updated along taking increasing values of $j$. It also keeps in memory the finite output word $o_j$ that has been output so far. For any $j$, if $\mathsf{dt}(x[{:}j])$ denotes the data that appear in $x$, the algorithm then decides, for each input $(\sigma, d) \in \Sigma \times (\mathsf{dt}(x[{:}j]) \cup \{\mathsf{d}_0\})$ whether $(\sigma, d)$ can safely be output, i.e., whether all accepting runs on words of the form $x[{:}j]y$, for an infinite word $y$, outputs at least $o_j(\sigma, d)$. The latter can be decided, given $T$, $o_j$ and $x[{:}j]$, by Lemma 17. Note that it suffices to look at data in $\mathsf{dt}(x[{:}j]) \cup \{\mathsf{d}_0\}$ only since, by definition of NRT, any data that is output is necessarily stored in some register, and therefore appears in $x[{:}j]$ or is equal to $\mathsf{d}_0$. Let us show that

**Algorithm 1:** Algorithm describing the machine $M_f$ computing $f$.

```
   Data: x ∈ dom(f)
1  o := ϵ ;
2  for j = 0 to ∞ do
3  |  for (σ, d) ∈ Σ × (dt(x[:j]) ∪ {d0}) do
4  |  |  if o.(σ, d) ⪯ f̂(x[:j]) then // such test is decidable by Lemma 17
5  |  |  |  o := o.(σ, d);
6  |  |  |  output (σ, d);
7  |  |  end
8  |  end
9  end
```

$M_f$ actually computes $f$. Let $x \in \mathrm{dom}(f)$. We have to show that the sequence $(M_f(x,j))_j$ converges to $f(x)$. Let $o_j$ be the content of variable $o$ of $M_f$ when exiting the inner loop at line 8, when the outer loop (line 2) has been executed $j$ times (hence $j$ input symbols have been read). Note that $o_j = M_f(x,j)$. We have $o_1 \preceq o_2 \preceq \ldots$ and $o_j \preceq \hat{f}(x[{:}j])$ for all $j \geq 0$. Hence, $o_j \preceq f(x)$ for all $j \geq 0$. To show that $(o_j)_j$ converges to $f(x)$, it remains to show that $(o_j)_j$ is non-stabilising, i.e. $o_{i_1} \prec o_{i_2} \prec \ldots$ for some infinite subsequence $i_1 < i_2 < \ldots$. First, note that $f$ being continuous is equivalent to the sequence $(\hat{f}(x[{:}k]))_k$ converging to $f(x)$. Therefore we have that $f(x) \wedge \hat{f}(x[{:}k])$ can be arbitrarily long, for sufficiently large $k$. Let $j \geq 0$ and $(\sigma, d) = f(x)[|o_j|+1]$. By the latter property and the fact that $o_j.(\sigma, d) \preceq f(x)$, necessarily, there exists some $k > j$ such that $o_j.(\sigma, d) \preceq \hat{f}(x[:k])$. Moreover, by definition of $\mathsf{NRT}$, $d$ is necessarily a data that appears in some prefix of $x$, therefore there exists $k' \geq k$ such that $d$ appears in $x[:k']$ and $o_j.(\sigma, d) \preceq \hat{f}(x[:k] \preceq \hat{f}(x[:k'])$. This entails that $o_j.(\sigma, d) \preceq o_{k'}$. So, we have shown that for all for all $j$, there exists $k' > j$ such that $o_j \prec o_{k'}$, which concludes the proof. □

Now that we have shown that computability is equivalent with continuity for functions defined by $\mathsf{NRT}$, we exhibit a pattern which allows to decide continuity. Such pattern generalises the one of [3] to the setting of data words, the difficulty lying in showing that our pattern can be restricted to a finite number of data.

**Theorem 19.** *Let $T$ be a functional* $\mathsf{NRT}$ *with $k$ registers. Then, for all $X \subseteq \mathcal{D}$ such that $|X| \geq 2k+3$ and $\mathsf{d}_0 \in X$, $T$ is not continuous at some $x \in (\Sigma \times \mathcal{D})^\omega$ if and only if $T$ is not continuous at some $z \in (\Sigma \times X)^\omega$.*

*Proof.* The right-to-left direction is trivial. Now, let $T$ be a functional $\mathsf{NRT}$ with $k$ registers which is not continuous at some $x \in (\Sigma \times \mathcal{D})^\omega$. Let $f : \mathrm{dom}([\![T]\!]) \to (\Gamma \times \mathcal{D})^\omega$ be the function defined by $T$, as: for all $u \in \mathrm{dom}([\![T]\!])$, $f(u) = v$ where $v \in (\Gamma \times \mathcal{D})^\omega$ is the unique data word such that $(u, v) \in [\![T]\!]$.

Now, let $X \subseteq \mathcal{D}$ be such that $|X| \geq 2k+3$ and $\mathsf{d}_0 \in X$. We need to build two words $u$ and $v$ labelled over $X$ which coincide on a sufficiently long prefix to allow for pumping, hence yielding a converging sequence of input data words whose images do not converge, witnessing non-continuity. To that end, we use a similar proof technique as for Theorem 9: we show that the language of interleaved runs whose inputs coincide on a sufficiently long prefix while their respective outputs mismatch before a given position is recognisable by an $\mathsf{NRA}$, allowing us to use the indistinguishability property. We also ask that one run presents sufficiently many occurrences of a final state $q_f$, so that we can ensure that there exists a pair of configurations containing $q_f$ which repeats in both runs.

On reading such $u$ and $v$, the automaton behaves as a finite automaton, since the number of data is finite ([15, Proposition 1]). By analysing the respective runs, we can, using pumping arguments, bound the position on which the mismatch appears in $u$, then show the existence of a synchronised loop over $u$ and $v$ after such position, allowing us to build the sought witness for non-continuity.

*Relabel over $X$* Thus, assume $T$ is not continuous at some point $x \in (\Sigma \times \mathcal{D})^\omega$. Let $\rho$ be an accepting run of $T$ over $x$, and let $q_f \in \inf(\mathsf{st}(\rho)) \cap F$ be an accepting state repeating infinitely often in $\rho$. Then, let $i \geq 0$ be such that for all $j \geq 0$, there exists $y \in \mathrm{dom}(f)$ such that $\|x \wedge y\| \geq j$ but $\|f(x) \wedge f(y)\| \leq i$. Now, define $K = |Q| \times (2k+3)^{2k}$ and let $m = (2i+3) \times (K+1)$. Finally, pick $j$ such that $\rho[1{:}j]$ contains at least $m$ occurrences of $q_f$. Consider the language:

$$L = \big\{\rho_1 \otimes \rho_2 \big| \|\mathsf{in}(\rho_1) \wedge \mathsf{in}(\rho_2)\| \geq j, \|\mathsf{out}(\rho_1) \wedge \mathsf{out}(\rho_2)\| \leq i \text{ and there are at least } m \text{ occurrences of } q_f \text{ in } \rho_1[1{:}j]\big\}$$

By Lemma 5, $L_\otimes(T)$ is recognised by an NRA with $2k$ registers. Additionnally, by Lemma 6, $M^i_j$ is recognised by an NRA with 2 registers. Thus, $L = L_\otimes(T) \cap O^{q_f}_{m,j} \cap M^i_j$, where $O^{q_f}_{m,j}$ checks there are at least $m$ occurrences of $q_f$ in $\rho_1[1{:}j]$ (this is easily doable from the automaton recognising $L_\otimes(T)$ by adding an $m$-bounded counter), is recognisable by an NRA with $2k+2$ registers.

Choose $y \in \mathrm{dom}(f)$ such that $\|x \wedge y\| \geq j$ but $\|f(x) \wedge f(y)\| \leq i$. By letting $\rho_1$ (resp. $\rho_2$) be an accepting run of $T$ over $x$ (resp. $y$) we have $\rho_1 \otimes \rho_2 \in L$, so $L \neq \varnothing$. By Proposition 4, $L \cap ((\Sigma \times X)^\omega \times (\Gamma \times X)^\omega) \neq \varnothing$. Let $w = \rho'_1 \otimes \rho'_2 \in L \cap ((\Sigma \times X)^\omega \times (\Gamma \times X)^\omega)$, $u = \mathsf{in}(\rho'_1)$ and $v = \mathsf{in}(\rho'_2)$. Then, $\|u \wedge v\| \geq j$, $\|f(u) \wedge f(v)\| \leq i$ and there are at least $m$ occurrences of $q_f$ in $\rho_1[1{:}j]$.

Now, we depict $\rho'_1$ and $\rho'_2$ in Figure 4, where we decompose $u$ as $u = u_1 \dots u_m \cdot s$ and $v$ as $v = u_1 \dots u_m \cdot t$; their corresponding images being respectively $u' = u'_1 \dots u'_m \cdot s'$ and $u'' = u''_1 \dots u''_m t''$. We also let $l = (i+1)(K+1)$ and $l' = 2(i+1)(K+1)$. Since the data of $u, v$ and $w$ belong to $X$, we know that $\tau_i, \mu_i : R \to X$.

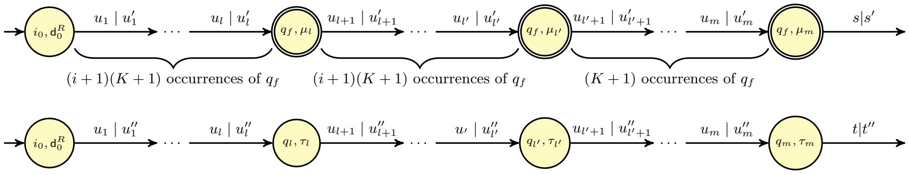

**Fig. 4.** Runs of $f$ over $u = u_1 \dots u_m \cdot s$ and $v = u_1 \dots u_m \cdot t$.

*Repeating configurations* First, let us observe that in a partial run of $\rho'_1$ containing more than $|Q| \times |X|^k$ occurrences of $q_f$, there is at least one productive transition, i.e. a transition whose output is $o \neq \varepsilon$. Otherwise, by the pigeonhole principle, there exists a configuration $\mu : R \to X$ such that $(q_f, \mu)$ occurs at least twice in the partial run. Since all transitions are improductive, it would mean that, by writing $w$ the corresponding part of input, we have $(q_f, \mu) \xrightarrow[T]{w|\varepsilon} (q_f, \mu)$. This partial run is part of $\rho'_1$, so, in particular, $(q_f, \mu)$ is accessible, hence by taking $w_0$ such that $(i_0, \tau_0) \xrightarrow[T]{w_0|w'_0} (q_f, \mu)$, we have that $f(w_0 w^\omega) = w'_0$, which is a finite word, contradicting our assumption that all accepting runs produce an infinite output. This implies that, for any $n \geq |Q| \times |X|^k$ (in particular for $n = l$), $\|u'_1 \dots u'_n\| \geq i+1$.

*Locate the mismatch* Again, upon reading $u_{l+1} \dots u_{l'}$, there are $(i+1)(K+1)$ occurrences of $q_f$. There are two cases:

(a) There are at least $i+1$ productive transitions in $\rho'_2$. Then, we obtain that $\|u''_1 \dots u''_{l'}\| > i$, so $\mathsf{mismatch}(u'_1 \dots u'_{l'}, u''_1 \dots u''_{l'})$, since we know $\|f(u) \wedge f(v)\| \leq i$ and they are respectively prefixes of $f(u)$ and $f(v)$, both of length at least $i+1$. Afterwards, upon reading $u_{l'+1} \dots u_m$, there are $K+1 > |Q| \times |X|^{2k}$ occurrences of $q_f$, so, by the pigeonhole principle, there is a repeating pair: there exist indices $p$ and $p'$ such that $l' \leq p < p' \leq m$ and $(q_f, \mu_p) = (q_f, \mu_{p'})$, $(q_p, \tau_p) = (q_{p'}, \tau_{p'})$. Thus, let $z_P = u_1 \dots u_p$, $z_R = u_{p+1} \dots u_{p'}$ and $z_C = u_{p'+1} \dots u_m \cdot t$ ($P$ stands for *prefix*, $R$ for *repeat* and $C$ for *continuation*; we use capital letters to avoid confusion with indices). By denoting $z'_P = u'_1 \dots u'_p$, $z'_R = u'_{p+1} \dots u'_{p'}$, $z''_P = u''_1 \dots u''_p$, $z''_R = u''_{p+1} \dots u''_{p'}$ and $z''_C = u''_{p'+1} \dots u''_m \cdot t''$ the corresponding images, $z = z_P \cdot {z_R}^\omega$ is a point of discontinuity. Indeed, define $(z_n)_{n \in \mathbb{N}}$ as, for all $n \in \mathbb{N}$, $z_n = z_P \cdot z_R^n \cdot z_C$. Then, $(z_n)_{n \in \mathbb{N}}$ converges towards $z$, but, since for all $n \in \mathbb{N}$, $f(z_n) = z''_P \cdot {z''_L}^n \cdot z''_C$, we have that $f(z_n) \not\xrightarrow[n\infty]{} f(z) = z'_P \cdot {z'_L}^\omega$, since $\mathsf{mismatch}(z'_P, z''_P)$.

(b) Otherwise, by the same reasoning as above, it means there exists a repeating pair with only improductive transitions in between: there exist indices $p$ and $p'$ such that $l \leq p < p' \leq l'$, $(q_f, \mu_p) = (q_f, \mu_{p'})$, $(q_p, \tau_p) = (q_{p'}, \tau_{p'})$, and $(q_f, \mu_p) \xrightarrow{u_{p+1} \dots u_{p'} | \varepsilon} (q_f, \mu_{p'})$, $(q_p, \tau_p) \xrightarrow{u_{p+1} \dots u_{p'} | \varepsilon} (q_{p'}, \tau_{p'})$. Then, by taking $z_P = u_1 \dots u_p$, $z_R = u_{p+1} \dots u_{p'}$ and $z_C = u_{p'+1} \dots u_m \cdot t$, we have, by letting $z'_P = u'_1 \dots u'_p$, $z'_R = u'_{p+1} \dots u'_{p'}$, $z''_P = u''_1 \dots u''_p$, $z''_R = \varepsilon$ and $z''_C = u''_{n'+1} \dots u''_m \cdot t''$, that $z = z_P \cdot {z_R}^\omega$ is a point of discontinuity. Indeed, define $(z_n)_{n \in \mathbb{N}}$ as, for all $n \in \mathbb{N}$, $z_n = z_P \cdot z_R^n \cdot z_C$. Then, $(z_n)_{n \in \mathbb{N}}$ indeed converges towards $z$, but, since for all $n \in \mathbb{N}$, $f(z_n) = z''_P \cdot z''_C$, we have that $f(z_n) \not\xrightarrow[n\infty]{} f(z) = z'_P \cdot {z'_R}^\omega$, since $\mathsf{mismatch}(z'_P, z''_P \cdot z''_C)$ (the mismatch necessarily lies in $z'_P$, since $\|z'_P\| \geq i+1$). □

**Corollary 20.** *Deciding whether an* NRT *defines a continuous function is* PSpace*-complete.*

*Proof.* Let $X \subseteq \mathcal{D}$ be a set of size $2k+3$ containing $\mathsf{d}_0$. By Theorem 19, $T$ is not continuous iff it is not continuous at some $z \in (\Sigma \times X)^\omega$, iff $[\![T]\!] \cap \big((\Sigma \times X)^\omega \times (\Gamma \times X)^\omega\big)$ is not continuous. By Proposition 3, such relation is recognisable by a finite transducer $T_X$ with $O(|Q| \times |X|^{|R|})$ states, which can be built on-the-fly. By [3], the continuity of functions defined by NFT is decidable in NLogSpace, which yields a PSpace procedure.

For the hardness, we reduce again from the emptiness problem of register automata, which is PSpace-complete [4]. Let $A$ be a register automaton over some alphabet $\Sigma \times \mathcal{D}$. We construct a transducer $T$ which defines a continuous function iff $L(A) = \varnothing$ iff the domain of $T$ is empty. Let $f$ be a non-continous function realised by some NRT $H$ (it exists by Example 16). Then, let $\# \notin \Sigma$ be a fresh symbol, and define the function $g$ as the function mapping any data word of the form $w(\#, d)w'$ to $w(\#, d)f(w')$ if $w \in L(A)$. The function $g$ is realised by an NRT which simulates $A$ and copies its inputs on the output to implement the identity, until it sees $\#$. If it was in some accepting state of $A$ before seeing $\#$, it branches to some initial state of $H$ and proceeds executing $H$. If there is some $w_0 \in L(A)$, then the subfunction $g_{w_0}$ mapping words of the form $w_0(\#, d)w'$ to $w_0(\#, d)f(w')$ is not continuous, since $f$ is not. Hence $g$ is not continuous. Conversely, if $L(A) = \varnothing$, then $\mathrm{dom}(g) = \varnothing$, so $g$ is continuous. □

In [3], non-continuity is characterised by a specific pattern (Lemma 21, Figure 1), i.e. the existence of some particular sequence of transitions. By applying this characterisation to the finite transducer recognising $[\![T]\!] \cap ((\Sigma \times X)^\omega \times (\Gamma \times X)^\omega)$, as constructed in Proposition 3, we can characterise non-continuity by a similar pattern, which will prove useful to decide (non-)continuity of test-free NRT in NLogSpace (cf Section 5):

**Corollary 21 ([3]).** *Let $T$ be an NRT with $k$ registers. Then, for all $X \subseteq \mathcal{D}$ such that $|X| \geq 2k+3$ and $\mathsf{d}_0 \in X$, $T$ is not continuous at some $x \in (\Sigma \times \mathcal{D})^\omega$ if and only if it has the pattern of Figure 5.*

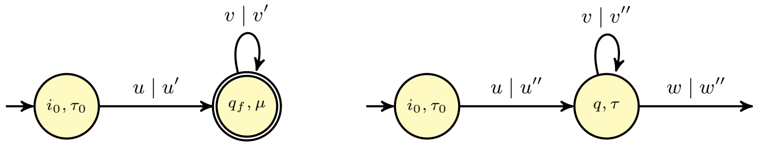

**Fig. 5.** A pattern characterising non-continuity of functions definable by an NRT: we ask that there exist configurations $(q_f, \mu)$ and $(q, \tau)$, where $q_f$ is accepting, as well as finite input data words $u, v$, finite output data words $u', v', u'', v''$, and an infinite input data word $w$ admitting an accepting run from configuration $(q, \tau)$ producing output $w''$, such that $\mathsf{mismatch}(u', u'') \vee (v'' = \varepsilon \wedge \mathsf{mismatch}(u', u''w''))$.

## 5 Test-free Register Transducers

In [7], we introduced a restriction which allows to recover decidability of the bounded synthesis problem for specifications expressed as non-deterministic register automata. Applied to transducers, such restriction also yields polynomial complexities when considering the functionality and computability problems.

An NRT $T$ is *test-free* when its transition function does not depend on the tests conducted over the input data. Formally, we say that $T$ is *test-free* if for all transitions $q \xrightarrow[T]{\sigma, \phi | \mathsf{asgn}, o} q'$ we have $\phi = \top$. Thus, we can omit the tests altogether and its transition relation can be represented as $\Delta' \subseteq Q \times \Sigma \times 2^R \times (\Gamma \times R)^* \times Q$.

*Example 22.* Consider the function $f : (\Sigma \times \mathcal{D})^\omega \to (\Gamma \times \mathcal{D})^\omega$ associating, to $x = (\sigma_1, d_1)(\sigma_2, d_2)\dots$, the value $(\sigma_1, d_1)(\sigma_2, d_1)(\sigma_3, d_1)\dots$ if there are infinitely many $a$ in $x$, and $(\sigma_1, d_2)(\sigma_2, d_2)(\sigma_3, d_2)\dots$ otherwise.

$f$ can be implemented using a test-free NRT with one register: it initially guesses whether there are infinitely many $a$ in $x$, if it is the case, it stores $d_1$ in the single register $r$, otherwise it waits for the next input to get $d_2$ and stores it in $r$. Then, it outputs the content of $r$ along with each $\sigma_i$. $f$ is not continuous, as even outputting the first data requires reading an infinite prefix when $d_1 \neq d_2$.

Note that when a transducer is test-free, the existence of an accepting run over a given input $x$ only depends on its finite labels. Hence, the existence of two outputs $y$ and $z$ which mismatch over data can be characterised by a simple pattern (Figure 6), which allows to decide functionality in polynomial time:

**Theorem 23.** *Deciding whether a test-free* NRT *is functional is in* PTIME.

*Proof.* Let $T$ be a test-free NRT such that $T$ is not functional. Then, there exists $x \in (\Sigma \times \mathcal{D})^\omega$, $y, z \in (\Gamma \times \mathcal{D})^\omega$ such that $(x, y), (x, z) \in [\![T]\!]$ and $y \neq z$. Then, let $i$ be such that $y[i] \neq z[i]$. There are two cases. Either $\mathsf{lab}(y[i]) \neq \mathsf{lab}(z[i])$, which means that the finite transducer $T'$ obtained by ignoring the registers of $T$ is not functional. By Proposition 8, such property can be decided in NLOGSPACE, so let us focus on the second case: $\mathsf{dt}(y[i]) \neq \mathsf{dt}(z[i])$.

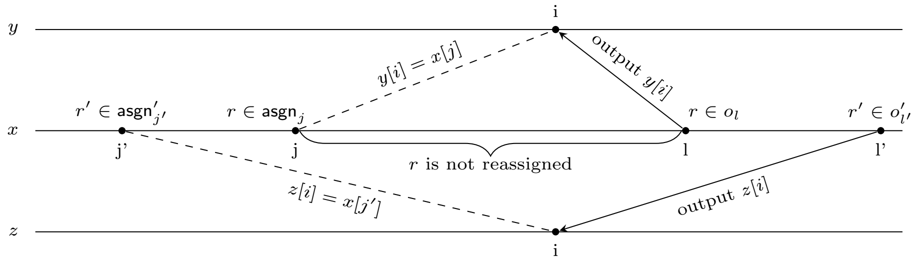

**Fig. 6.** A situation characterising the existence of a mismatch over data. Since acceptance does not depend on data, we can always choose $x$ such that $\mathsf{dt}(x[j]) \neq \mathsf{dt}(x[j'])$. Here, we assume that the labels of $x, y$ and $z$ range over a unary alphabet; in particular $y[i] = x[j]$ iff $\mathsf{dt}(y[i]) = \mathsf{dt}(x[j])$. Finally, for readability, we did not write that $r'$ should not be reassigned between $j'$ and $l'$. Note that the position of $i$ with regards to $j, j', l$ and $l'$ does not matter; nor does the position of $l$ w.r.t. $l'$.

We here give a sketch of the proof: observe that an input $x$ admits two outputs which mismatch over data if and only if it admits two runs which respectively store $x[j]$ and $x[j']$ such that $x[j] \neq x[j']$ and output them later at the same output position $i$; the outputs $y$ and $z$ are then such that $\mathsf{dt}(y[i]) \neq \mathsf{dt}(z[i])$. Since $T$ is test-free, the existence of two runs over the same input $x$ only depends on its finite labels. Then, the registers containing respectively $x[j]$ and $x[j']$ should not be reassigned before being output, and should indeed output their content at the same position $i$ (cf Figure 6). Besides, again because of test-freeness, we can always assume that $x$ is such that $x[j] \neq x[j']$. Overall, such pattern can be checked by a 2-counter Parikh automaton, whose emptiness is decidable in PTIME [8] (under conditions that are satisfied here). □

Now, let us move to the case of continuity. Here again, the fact that test-free NRT conduct no test over the input data allows to focus on the only two registers that are responsible for the mismatch, the existence of an accepting run being only determined by finite labels.

**Theorem 24.** *Deciding whether a test-free* NRT *defines a continuous function is in* PTIME.

*Proof.* Let $T$ be a test-free NRT. First, it can be shown that $T$ is continuous if and only if $T$ has the pattern of Figure 7, where $r$ is coaccessible (since acceptance only depends on finite labels, $T$ can be trimmed[3] in polynomial time).

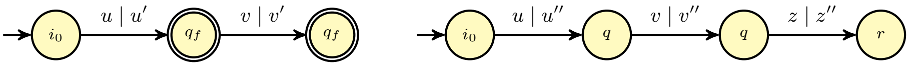

**Fig. 7.** A pattern characterising non-continuity of functions defined by NRT, where we ask that there exist some states $q_f$, $q$ and $r$, where $q_f$ is accepting, as well as finite input data words $u, v, z$ and finite output data words $u', v', u'', v'', z''$ such that $\mathsf{mismatch}(u', u'') \vee (v'' = \varepsilon \wedge \mathsf{mismatch}(u', u''z''))$. Register assignments are not depicted, as there are no conditions on them. We unrolled the loops to highlight the fact that they do not necessarily loop back to the same configuration.

Now, it remains to show that such simpler pattern can be checked in PTIME. We treat each part of the disjunction separately:

(a) there exists $u, u', u'', v, v', v''$ s.t. $i_0 \xrightarrow{u|u'} q_f \xrightarrow{v|v'} q_f$ and $i_0 \xrightarrow{u|u''} q \xrightarrow{v|v''} q$, where $q_f \in F$ and $\mathsf{mismatch}(u', u'')$. Then, as shown in the proof of Theorem 23, there exists a mismatch between some $u'$ and $u''$ produced by the same input $u$ if and only if there exists two runs and two registers $r$ and $r'$ assigned at two distinct positions, and later on output at the same position. Such pattern can similarly be checked by a 2-counter Parikh automaton; the only difference is that here, instead of checking that the two end states are coaccessible with a common $\omega$-word, we only need to check that $q_f \in F$ and that there is a synchronised loop over $q_f$ and $q$, which are regular properties that can be checked by the Parikh automaton with only a polynomial increase.

(b) there exists $u, u', u'', v, v', z, z''$ s.t. $i_0 \xrightarrow{u|u'} q_f \xrightarrow{v|v'} q_f$ and $i_0 \xrightarrow{u|u''} q \xrightarrow{v|\varepsilon} q \xrightarrow{z|z''} r$, where $q_f \in F$ and $\mathsf{mismatch}(u', u''z'')$. By examining again the proof of Theorem 23, it can be shown that to obtain a mismatch, it suffices that the input is the same for both runs only up to position $\max(j, j')$. More precisely, there is a mismatch between $u'$ and $u''z''$ if and only if there exists two registers $r$ and $r'$ and two positions $j, j' \in \{1, \dots, \|u\|\}$ such that $j \neq j'$, $r$ is stored at position $j$, $r'$ is stored at position $j'$, $r$ and $r'$ are respectively output at input positions $l \in \{1, \dots, \|u\|\}$ and $l' \in \{1, \dots, \|uz\|\}$ and they are not reassigned in the meantime. Again, such property, along with the fact that $q_f \in F$ and the existence of a synchronised loop can be checked by a 2-counter Parikh automaton of polynomial size.

Overall, deciding whether a test-free NRT is continuous is in PTIME. □

[3] We say that $T$ is trim when all its states are both accessible and coaccessible.
